# The Georgia Medical Association

**Published:** 1892-05

**Authors:** 


### Editorial.

### THE GEORGIA MEDICAL ASSOCIATION.

The forty-third annual session of the Medical Association of Georgia was held in Columbus, April 20th, 21st and 22d. The meeting proved the most successful in the entire history of the organization, over two hundred members being present, with an addition of sixty-one new members.

A large number of very interesting papers were read, which were ably discussed.

Columbus deserves much credit for the manner in which her guests were entertained. On the evening of the 21st an elegant banquet was tendered the visiting doctors at the Rankin House by the physicians and citizens of Columbus. The menu was most delicious and the responses to the several toasts quite happy. Following is the order in which they came:

The Medical Association of Georgia—Dr. T. M. McIntosh.
Woman—Dr. W. F. Westmoreland.
The Law—Hon. Lionel C. Levy.
The Press—Col. B. H. Richardson.
Practice of Medicine—Dr. Seth N. Jordan.
Our Past Presidents—Dr. A.W. Griggs.
The City of Columbus—Mayor J. J. Slade.
The Country Doctor—Dr. Frank Ridley.
Medicine as a Science—Dr. J. McFadden Gaston.

Being crowded for space we are permitted to publish only one of the toasts in this issue, but some of the others will follow at a subsequent date. We present in part the response of Dr. W. F. Westmoreland, of Atlanta, whose subject was

WOMAN.

"Here's to her health
Whose heart is wealth
Whose lips life's loveliest flower;
Whose cheeks are fair
As lilies are
Beneath the gentle shower."

Oh, woman, woman, what a part you play in our lives! What a trust we should have in thee! Even God showed the infinite trust he placed in you, when he conferred on you the divine obligation of perpetuating those made after his own image.

We cannot speak of woman without thinking of the associations of love by which our lives are bound to them from the beginning to the end.

Among the earliest recollections of our life, none of us will ever forget the tender eyes loved so well, that looked into ours with an expression of infinite love and sympathy. This was in our childhood's happy day, when with loving care she guided our tottering footsteps. How blest we have been rendered by that love. And the beautiful flowers of affection she planted along the pathway of our lives, are the brightest ones we gather into a wreath as we approach our journey's end. As we grow older, as we approach manhood's estate, another love comes into our lives, and the protecting love of the past is overshadowed by another, for the inspirations of the heart are alike everywhere. You forget that nothing will ever come into your life any truer or more honest than the regard, interest and love which a mother feels for you.

Now begins the Spring day of life, the day when we realize that we have found the woman that we wish to be everything to us—friend, comrade, counsellor, guide, sweetheart, wife—the one thing we think absolutely necessary to our existence. This is the love which is the flower of youth. These are the days of happy anticipation to be crowned by the sweetest realization. These days when we can see her, hear her voice, and touch her hand, our hearts have a feeling of perfect content and the world seems flooded with sunshine.

The sunshine seems so warm and caressing, the sky so divinely blue, the violets so purple and sweet.

We wonder if it is the day that is so divine.

Ah! love is a heavenly blessing, for a thing so perfect and ennobling must be like the beauties of nature, God's handiwork.

But then the scene changes and we reach that age where we are just as dependent upon their mercy now as we were upon their love in the tender days of youth.

These are the days when we sit on the opposite side of the hearth, and as we look into each other's eyes we realize that the pallor of evening twilight, instead of the morning of the beautiful day of the past is dawning upon us.

But then there may be a different aspect—she may have passed away from the scene, but after all, I should think that the tenderest faith to devote your life to, would be memories which had so entwined themselves around your heart-strings, that after the acute pain of the lost had died away (with the lapse of time), your whole idea would be living the life they would have wished, ever striving for higher things.

Then such memory vibrating through the heart, touching the trembling living strings in the tenderest manner, would recall the lingering memory of sweet sympathy given in the past, and that even now you feel extending beyond the grave. But after all, give me the living memory, the living association, even though it sometimes may be one-sided. Then when the path-way of life becomes dark and our footsteps tottering, I will recall the lines :

In the desert a fountain is springing,
In the wide waste there still is a tree,
And a bird in the solitude singing,
Which speaks to my spirit of thee.

